# Neuroblastoma treatment in the post-genomic era

**DOI:** 10.1186/s12929-017-0319-y

**Published:** 2017-02-08

**Authors:** Maria Rosaria Esposito, Sanja Aveic, Anke Seydel, Gian Paolo Tonini

**Affiliations:** 1 Paediatric Research Institute, Fondazione Città della Speranza, Neuroblastoma Laboratory, Corso Stati Uniti, 4, Padua, 35127 Italy; 2 Department of Biology, University of Padua, Padua, Italy

**Keywords:** Neuroblastoma, Omics, Personalised medicine, Targeted therapy

## Abstract

Neuroblastoma is an embryonic malignancy of early childhood originating from neural crest cells and showing heterogeneous biological, morphological, genetic and clinical characteristics. The correct stratification of neuroblastoma patients within risk groups (low, intermediate, high and ultra-high) is critical for the adequate treatment of the patients.

High-throughput technologies in the Omics disciplines are leading to significant insights into the molecular pathogenesis of neuroblastoma. Nonetheless, further study of Omics data is necessary to better characterise neuroblastoma tumour biology. In the present review, we report an update of compounds that are used in preclinical tests and/or in Phase I-II trials for neuroblastoma. Furthermore, we recapitulate a number of compounds targeting proteins associated to neuroblastoma: MYCN (direct and indirect inhibitors) and downstream targets, Trk, ALK and its downstream signalling pathways. In particular, for the latter, given the frequency of *ALK* gene deregulation in neuroblastoma patients, we discuss on second-generation ALK inhibitors in preclinical or clinical phases developed for the treatment of neuroblastoma patients resistant to crizotinib.

We summarise how Omics drive clinical trials for neuroblastoma treatment and how much the research of biological targets is useful for personalised medicine. Finally, we give an overview of the most recent druggable targets selected by Omics investigation and discuss how the Omics results can provide us additional advantages for overcoming tumour drug resistance.

## Background

Neuroblastoma is an embryonal malignancy of early childhood of the sympathetic nervous system belonging to the neuroblastic tumors that also include ganglioneuroblastoma and ganglioneuroma. The nosologic group of neuroblastoma is very heterogeneous in terms of biologic, genetic, clinical and morphologic characteristics [1, 2]. Neuroblastoma presents with a poor prognosis for individuals diagnosed at over 18 months of age with disseminated disease as metastatic processes in liver, bone marrow, skin and several other organs [3]. The highly heterogeneous clinical behaviour of disease makes the prediction of the patient’s individual risk at the time of diagnosis the major goal in choosing an adequate therapeutic approach. Many efforts done by performing the so called “Omics” technologies have shed light on the biology of this tumour allowing more accurate stratification of the patients in proper risk group.

In fact, by combining the results of Omics data and available clinical/biological parameters, the International Neuroblastoma Risk Group (INRG) task force has established a stratification system of neuroblastoma patients taking into consideration diverse prognostic factors (i.e., clinical stage, patient’s age at diagnosis, tumour histology (Shimada system) [4], grade of tumour differentiation, *MYCN* oncogene amplification, 11q deletion and DNA ploidy). Based on these criteria, neuroblastoma patients are currently subdivided into (very) low-, intermediate-, high- and ultra-high-risk groups. Nowadays, about half of all diagnosed cases are classified as high-risk (HR) for disease relapse, while overall survival rates still show only modest improvement, less than 40% at 5 years [5],. Therefore, recent discoveries regarding the understanding of the genetic basis of neuroblastoma and Omics data should necessarily be integrated in current knowledge of this malignancy in order to assure more accurate diagnosis for each patient and ascertain a good medical practice in terms of personalised therapy. In this regard, the awareness of the sequence of the entire human genome and the development of high-throughput Omics technologies has changed the approach to study neuroblastoma. Genome-wide information of amplifications and deletions of genomic regions, or somatically acquired genetic variations, common predisposing genetic variants and mRNA expression profiles have greatly helped us in better understanding of tumour behaviour. In this review we provide an overview on recent Omics studies, and how they direct current and future therapeutic approaches, shaping in that way the clinical trials set for neuroblastoma patients.

## Therapeutic solutions to approach the treatment of neuroblastoma

### Immunotherapy

The HR patients require very intensive treatments, including chemotherapy, surgery, radiotherapy, myeloablative chemotherapy with stem cell rescue, immunotherapy with anti-GD2 (disialoganglioside, tumour-associated surface antigen) antibody and differentiation therapy with 13-cis retinoic acid. However, new clinical trials for HR neuroblastoma patients are ongoing: i) a phase III trial that demonstrated significant improvement in event-free survival after combined immunotherapy with granulocyte-macrophage colony-stimulating factor GM-CSF, IL-2 and the ch14.18 anti-GD2 antibody (NCT00026312; list of all clinical trials discussed here can be found in Table 1) [6]; ii) a phase III randomized study (SIOPEN) for isotretinoin (13-cis-RA) and ch14.18 efficacy testing, in combination or not with IL-2 and after autologous stem cell transplantation (NCT01704716) [7]; and iii) two trials using L1-cell adhesion molecule (L1-CAM) together with GD2-specific chimeric antigen receptors (CARs) to demonstrate anti-tumour activity in intensely treated relapsed or refractory neuroblastoma patients (NCT01822652) [8]. The results of the listed trials are expected in 2017 and onwards.

**Table 1:** Drugs of clinical trials for HR neuroblastoma interventetion

ClinicalTrials ID	Original study	Phase	Status	Reference^a^
NCT00026312	Isotretinoin With or Without Dinutuximab, Aldesleukin and Sargramostim Following Stem Cell Transplant in Treating Patients With Neuroblastoma	phase III	Completed	Yu AL et al., 2010 [6]
NCT01704716	High Risk Neuroblastoma Study 1.7 of SIOP-Europe (SIOPEN)	phase III	Recruiting	Dobrenkov K & Cheung NK, 2014 [7]
NCT01822652	3rd Generation GD-2 Chimeric Antigen Receptor and iCaspase Suicide Safety Switch, Neuroblastoma, GRAIN	phase I	Active, not recruiting	Heczey A & Louis CU, 2013 [8]
NCT02395666	Preventative Trial of Difluoromethylornithine (DFMO) in High Risk Patients With Neuroblastoma That is in Remission	Phase 2	Active, not recruiting	Wallick CJ et al., 2005 [64]
NCT01586260	Preventative Trial of DFMO in Patients With High Risk Neuroblastoma in Remission	Phase 2	Active, not recruiting	Wallick CJ et al., 2005 [64]
NCT01059071	Safety Study for Refractory or Relapsed Neuroblastoma With DFMO Alone and in Combination With Etoposide	Phase 1	Completed	Wallick CJ et al., 2005 [64]
NCT02097810	Study of Oral RXDX-101 in Adult Patients With Locally Advanced or Metastatic Cancer Targeting NTRK1, NTRK2, NTRK3, ROS1 or ALK Molecular Alterations	phase I	Recruiting	Lee J et al., 2015 [83]
NCT01742286	Phase I Study of LDK378 in Pediatric, Malignancies With a Genetic Alteration in Anaplastic Lymphoma Kinase (ALK)	phase I	Recruiting	Schulte JH et al., 2013 [69]
NCT01871805	A Study of CH5424802/RO5424802 in Patients With ALK-Rearranged Non-Small Cell Lung Cancer	phase II	Active, not recruiting	McKeage K, 2015 [86]
NCT01049841	Perifosine With Temsirolimus for Recurrent Pediatric Solid Tumors	phase I	Active, not recruiting	Rodrik-Outmezguine VS et al., 2011 [104]
NCT01767194	Irinotecan Hydrochloride and Temozolomide With Temsirolimus or Dinutuximab in Treating Younger Patients With Refractory or Relapsed Neuroblastoma	Phase 2	Recruiting	Geoerger B et al., 2012 [105]

^a^ References are cited in review manuscript

### Targeting MYCN

For more than 30 years, *MYCN* status (amplified versus single copy) has been determined to be one of the strongest biological markers for neuroblastoma, providing a negative prognosis for a subset of patients with amplified *MYCN* [9–12]. Since a discovery of a correlation between *MYCN*, rapid tumour progression and poor prognosis of neuroblastoma patients, many efforts have been made in developing suitable MYCN drug that could impair its functions, and the same attempts are still ongoing. This is because of difficulties in developing an optimal therapy against MYCN due to a lack of appropriate surfaces on its DNA-binding domain to which drugs can bind. This problem persists not only for MYCN but also for other Myc family members [13]. Therefore, at present, a more widely accepted approach for MYCN regulation involves its indirect targeting [14].

#### Indirect targeting of MYCN expression and function

A number of compounds currently in use for the cure of neuroblastoma patients have been tested for their capacity to down-regulate MYCN expression. Among these compounds are retinoic acid [15] and other MYCN non-specific inhibitors such as HDAC inhibitors [16, 17] or inhibitors of the PI3K/AKT/mTOR pathway [18, 19]. The capacity of these compounds to down-regulate MYCN expression has been confirmed, but their effectiveness is variable. Therefore, other strategies have been adopted to target MYCN indirectly, by altering the function of other proteins known to regulate MYCN protein stability or by manipulating downstream targets of MYCN [20, 21].

#### Aurora A and Aurora B inhibitors

**Fig. 1:**
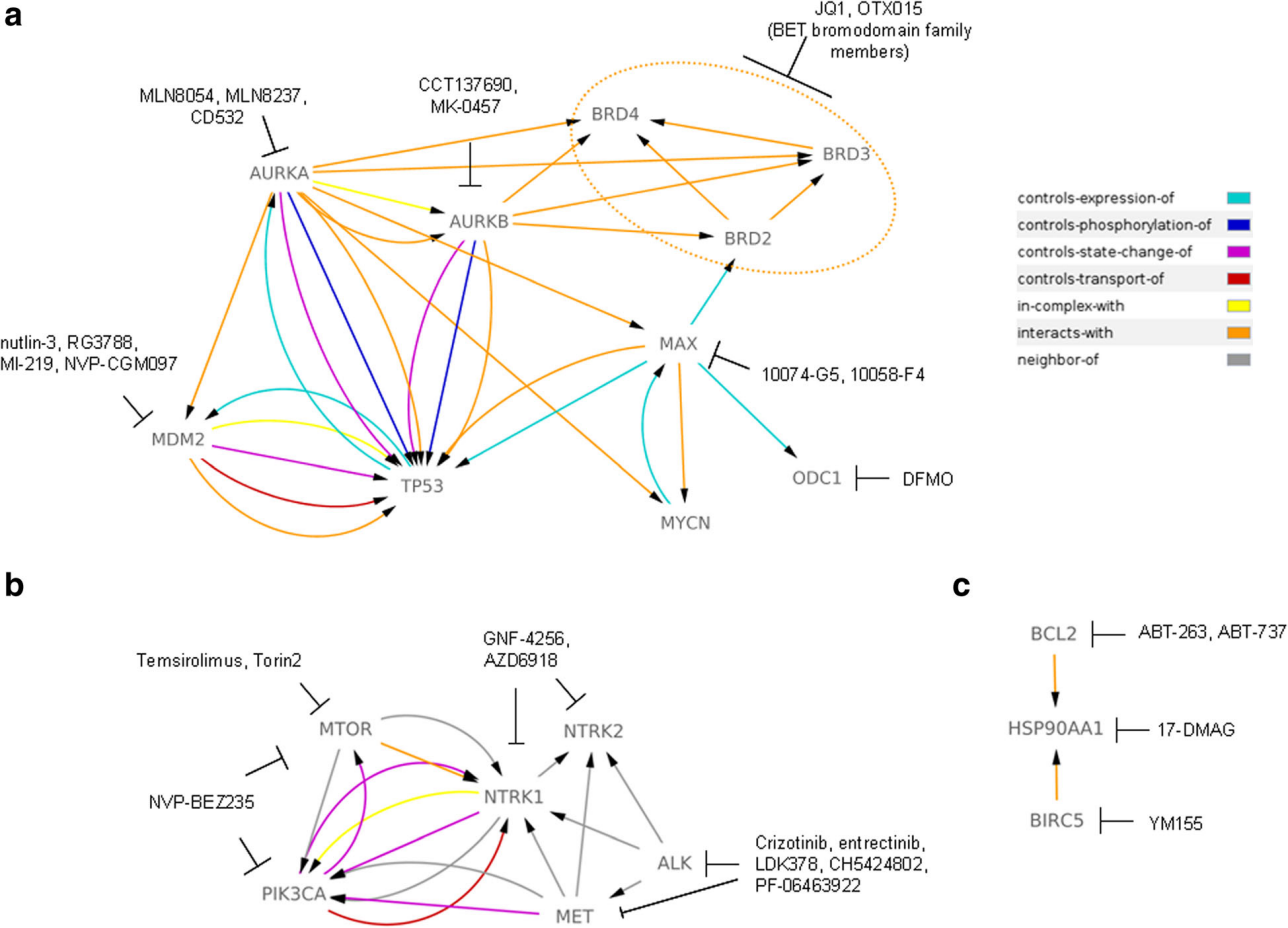
Schematic presentation of current pre-clinically tested drugs in neuroblastoma. A discussed anti-tumor drugs used against neuroblastoma in vitro and/or in vitro, and their targets are presented. In addition, a connection between the molecular targets is determined by the *arrows*. Legend shows a type of interaction described between the molecules **a** Indicates targeting of MYCN and P53/MDM dependent pathways. **b** Depicts drugs against ALK, Trk and PI3K/AKT/mTOR pathway. **c** Illustrates a targeting of main anti-apoptotic molecules. *Gene* symbol and its corresponding protein: *NTRK1* – TrkA; *NTRK2* – TrkB; *PIK3CA* - PI3K, *BIRC5* – Survivin (Data resource: http://www.pathwaycommons.org/)

The serine/threonine kinases Aurora A (AURKA) and Aurora B (AURKB) are crucial regulators of the cell cycle. Their coding genes differ in subcellular distribution and the protein products in their specific functions [22]. AURKA stabilizes MYCN through a direct protein-protein interaction, making MYCN less degradable by the proteasome [23]. *AURKA* mRNA expression has been described as a negative prognostic factor for neuroblastoma patients [24]. Therefore, AURKA has garnered much interest as a target in this disease [24]. On the other side, AURKB has been confirmed as a direct transcriptional target of MYCN, and its expression was observed increased in patients with poor outcomes [25]. Both kinases are therefore candidates for successful targeting with specific inhibitors. In fact, many preclinical studies have been conducted with anti-AURKA compounds. Among these compounds are orally active small-molecule inhibitors of AURKA (Fig. 1a), MLN8054 and MLN8237 (alisertib) [3, 26]. Both compounds have been tested in vitro and in vivo. However, of these two compounds, particular interest was given to MLN8237 due to its higher potency to inhibit AURKA, whereas dose-limiting toxicity was observed for MLN8054 [27, 28]. Nevertheless, the therapeutic promise of MLN8237 that was previously observed in vitro was not confirmed when tested in neuroblastoma patients, since it showed low efficacy, particularly in neuroblastoma patients with *MYCN*-amplification [29].

An interesting screening approach for the evaluation of the most potent inhibitors of AURKA has been proposed at the preclinical level by Gustafson and colleagues [30]. Their principal aim was to select a candidate compound that would lead to the degradation of the MYCN protein. The authors wanted to create an AURKA inhibitor able to compromise protein conformation and hence perturb MYCN-AURKA interaction [23]. Starting from tozasertib as a chemical model, the authors selected the candidate CD532 as a strong inhibitor of AURKA, which fulfilled the desired function of MYCN protein destabilisation. Application of CD532 induced an inactive AURKA conformation that provoked loss of MYCN protein due to its degradation [31]. Tested in vitro or in vivo using a MYCN-amplified neuroblastoma xenograft model, CD532 showed remarkable features in eradicating MYCN protein, warranting its probable use against neuroblastoma in future therapies. We are expecting an optimised version of CD532, which will allow its application in clinical trials.

Another approach applicable to the therapy of neuroblastoma patients is targeting of both aurora kinases, using non-selective anti-aurora compounds. In fact, pan-aurora kinase inhibitors are a subject of interest of many researchers who believe in their potency as anti-tumour drugs. By affecting both Auroras, A and B, a more substantial impact on tumour cells might be expected. To date, the pan-aurora inhibitors CCT137690 [32] and tozasertib (VX-680, MK-0457) [33] have been tested. Each of them has been demonstrated as a potential drug for targeting drug-resistant neuroblastoma cells [34], which has made them interesting candidates for further clinical evaluation.

#### Inhibitors of MYCN/MAX interaction

As other members of the MYC family of proto-oncogenes, MYCN also works as a transcriptional activator. To fulfil this action, MYCN requires the formation of a heterodimer with the MAX protein [35]. This binding is necessary for proper activity of the MYCN protein; hence, its obstruction has been considered as a strategy through which MYCN can be targeted in tumours. For this purpose, several small molecules have been designed for MYC inhibition and have been proven as efficient blockers of MYCN/MAX interactions. Among them are the structurally unrelated compounds 10074-G5 and 10058-F4 (Fig. 1a), which have been tested in vitro and which produce satisfying effects on neuronal differentiation and the induction of apoptosis [36]. Whether these compounds can repeat their effectiveness against neuroblastoma cells in vivo still remains to be verified.

#### Bet inhibitors

Another well-accepted approach for indirect MYCN-targeting is by inhibiting the BET (bromodomain and extra-terminal domain) family of proteins, which are important for transcriptional regulation of many genes including MYCN. One of the compounds developed for this purpose is the small molecule BET bromodomain inhibitor JQ1. Puissant and colleagues [37] demonstrated the use of JQ1 as a promising strategy for blocking the growth of MYCN-dependent neuroblastoma cells in vitro. It has been confirmed that JQ1 has anti-tumour properties in vivo, suggesting that JQ1 might be an option for the treatment of MYCN-dependent neuroblastomas [38]. However, additional studies are necessary to confirm JQ1’s effectiveness in the clinical setting. More recently, a European-American collaboration is applying BET inhibitors in neuroblastoma therapy [39]. In this study, Henssen et al. evaluated OTX015 as a promising anti-tumour drug in MYCN-driven neuroblastomas. In particular, OTX015 was shown to have a potent inhibitory effect on the growth of either mouse or human MYCN-dependent neuroblastomas. The mechanism of action involves the impediment of BRD4, one of the BET family proteins, to maintain active transcription of genes with super enhancers in their promoter regions. Interestingly, *MYCN* is among the genes that have super enhancers. Taken together, the data from the latter report suggest that OTX015 is a reasonable choice for targeted therapy of MYCN-amplified neuroblastomas.

#### MYCN downstream pathway targeting

It is possible that targeting of the proteins in the pathways downstream of MYCN might be also an useful and strategic alternative to direct inhibition of MYCN. There are several targetable candidates downstream of MYCN for which drugs are already available: MDM2 (by nutlin-3 or RG3788) [40], ODC1 (by difluoromethylornithine -DFMO) [41] and mTOR (by Temsirolimus) [42].

##### P53/MDM2 targeting

Unlike tumours in adults, which tend to overcome physiological regulation of P53 tumor-supressor by the means of mutations of *TP53* gene, neuroblastoma is rarely associated with those mutations [43]. Nonetheless, the P53 pathway is often impaired in childhood cancers because of upstream P53/MDM2/P14^ARF^ network aberrations. Therefore, it is of great interest to understand the interaction between P53 and its main negative regulator MDM2, as it may lead towards a therapeutic approach in paediatric patients with malignancies that do not have *TP53* mutations and who have poor prognoses [44]. In neuroblastoma, however, there is evidence that the P53 pathway is inactivated [45], and the inactivation of the P53 pathway occurs mainly at the time of relapse, probably contributing to chemoresistance. Several studies have confirmed that wild-type *TP53* alleles exist in most cases of newly diagnosed neuroblastoma, but after chemotherapy, the P53/MDM2/P14^ARF^ pathway is repressed, in part because of the abnormal inhibition of P53 by MDM2 [46–48]. This finding suggests that down-regulation of the P53 axis may underlie the treatment of patients who acquire drug resistance, which is a situation that is frequently observed in HR neuroblastoma. Although P53 is very rarely mutated in primary neuroblastoma at diagnosis and its downstream effectors are functional [47], multiple hits seem to cooperate to impair P53 functions, including deregulation of the ARF/MDM2 pathway [49, 50], expression of microRNAs that can target P53 pathways [51], and repression of P53-mediated autophagy [52]. Recent studies are focusing on current therapies and novel drugs targeting P53 signalling in neuroblastoma to understand the equilibrium between P53 family proteins and their regulation in neuroblastoma [53].

As so, a very frequent functional abnormalities detected in the P53/MDM2/p14^ARF^ pathway of relapsed patients shed light on its potential clinical targeting. One of the strategies to affect this pathway is by perturbing the P53/MDM2 interaction, in which MDM2 acts as a negative regulator of P53 levels [54]. Small molecules, such as nutlin-3 or MI-219, can interact with MDM2 by mimicking the P53 N-terminal region, where MDM2 binds to P53. Both of these small molecules have been tested in neuroblastoma, and the results of the studies showed that the effects depend on the MYCN status of neuroblastoma cells [21, 55]. More precisely, it has been found that overexpression of MYCN sensitises neuroblastoma cells to the use of MDM inhibitors, confirming that MYCN and MDM2 together confer pro-survival benefits to tumour cells [56]. Regarding nutlin-3, it has been reported to work independently of P53, affecting other important pro-tumour molecules, such as P73 or multidrug resistance protein 1 (MDR-1), that are responsible for drug resistance in different types of cancer [57]. Tests of MDM2–P53 antagonists are ongoing in several clinical trials in which these antagonists are administered either alone or in combination with other anti-cancer drugs [58]. We will have to wait and see the outcome of these trials to draw a conclusion about the promise of these inhibitors for use in personalised targeting. Until then, a strategy that might be adopted for the selection of the patients who might benefit from treatment with these compounds was suggested by Jeay et al. [59]. The authors described a gene signature that enables rapid prediction of tumours sensitive to NVP-CGM097, a potent and selective MDM2 inhibitor [60]. The same approach could be used for the recruitment of neuroblastoma patients for whom inhibition of P53/MDM2 might be highly effective.

##### ODC1

Encodes for ornithine decarboxylase 1, an enzyme required for synthesis of polyamines. The level of this enzyme is increased in highly metabolically active cells, such are the normal growing cells, but also transformed neuroblasts. In fact, the *MYCN*-driven neuroblastomas promote polyamine production by coordinating its downstream targets among which *ODC1* [61]. Therefore, a targeting of polyamine metabolism in *MYCN*-positive neuroblastoma has been considered preclinically and also during clinical trials [61, 62]. The efficiency of an irreversible inhibitor of the ODC1, known as difluoromethylornithine (DFMO; Eflornithine), drew a particular attention of oncologists [63]. It has been confirmed that the pre-emptive block of polyamine production by DFMO could impair tumour growth either in vitro or in the in vivo *MYCN*-mouse model [64]. These findings support not only the relevance of MYCN for the synthesis of polyamines, but also imply that depletion of this metabolic route might be a successfully alternative to direct MYCN targeting in neuroblastoma patients. In the moment, DFMO is tested either alone or together with other chemotherapics (NCT02395666, NCT01586260 and NCT01059071 - Table 1) and results of clinical trials are expecting.

##### mTOR

Mammalian target of rapamycin plays an essential function in cells’ growth regulation and protein production control [65]. Targeting of mTOR is very attractive since its block leads to MYCN destabilization, unfavouring therefore neuroblastoma growth [66]. However, since mTOR signals downstream from PI3K/AKT pathway, its targeting will be discussed together with drugs against this signalling branch.

### Inhibitors of anaplastic lymphoma kinase (ALK)

ALK is a receptor tyrosine kinase (RTK) implicated in the development of neuroblastoma [67–69]. As discussed previously [70], activating mutations in the *ALK* gene have been described in either familial neuroblastoma (under 1%) or in sporadic disease (approximately 8%) [71, 72]. Additionally, ALK has been confirmed as a target of the MYCN transcription factor, which automatically links this molecular marker with a poor outcome in neuroblastoma patients. Therefore, it is not surprising that retinoic acid can down-regulate the expression of the *ALK* gene as well, as a direct consequence of *MYCN* down-regulation [73]. Scientists interested in ALK share a strong confidence in its targeting during anti-neuroblastoma treatment. In fact, many of them believe that inhibition of ALK could ensure improved outcomes for neuroblastoma patients. Therefore, many strategies have been adopted in blocking the constitutive activation of ALK [74]. Because ALK is a cell-membrane receptor, its use in antibody-targeted therapy has been considered. This possibility was tested by the use of antibodies that block conformational activation of the tyrosine kinase domain after dimerization of two nearby ALK receptors [75]. However, this approach showed certain limitations, which might be improved by combining ALK-targeted immunotherapy with next-generation ALK inhibitors that act intracellularly [76].

#### Novel ALK inhibitors

A new generation of anti-ALK compounds inhibit kinase activity of this RTK. These compounds recognize and bind to the adenosine triphosphate (ATP) pocket of the receptor. Thus, the compounds compete with ATP, thereby preventing subsequent autophosphorylation, which is necessary for further signal transduction. Many ALK inhibitors have been tested either preclinical or clinically with a wide range of effectiveness. The most known anti-ALK drug is crizotinib (Pfizer; Fig. 1b), which gave promising results during treatment of patients with deregulated ALK function [77, 78]. This drug is a small molecule inhibitor capable of targeting ALK, ROS1 and MET RTKs. In vitro studies demonstrated that crizotinib is particularly efficient in neuroblastoma cells with the R1275Q mutation. Hence, crizotinib might be a valuable choice for the treatment of neuroblastoma patients with either amplifications or mutations in the *ALK* gene [79]. One point of caution is that we might need to use crizotinib in combination with other drugs in order to prevent resistance phenomena [80]. This hypothesis is in line with recent results published by Krytska and colleagues [81], who confirmed that when used in combination with the current chemotherapeutic agents topotecan and cyclophosphamide, crizotinib exhibited increased cytotoxic effects. Interestingly, deep sequencing has been shown to be an efficient approach for quick detection of *ALK* mutations within tumour biopsies responsible for resistance to crizotinib [82]. This technique might be useful for follow-up assessments of treatment efficacy by allowing the detection of possible resistance long before it actually develops. Another newly proposed ALK inhibitor is entrectinib (Ignyta Inc) which is currently being tested in a clinical trial (NCT02097810 – Table 1) [83]. This drug showed excellent cytotoxic effects in vitro, particularly in neuroblastoma cells with amplified *ALK* [84]. Additionally, the activity of entrectinib against neuroblastoma cells bearing *ALK* mutations was significantly improved when this drug was combined with chloroquine. This proposed combination was justified by the findings that application of entrectinib induced autophagy that protected tumour cells from death. In this work, a similar behaviour was observed for crizotinib, which induced autophagy in neuroblastoma cells when tested under the same in vitro conditions. Besides affecting ALK, entrectinib was also confirmed as an effective and promising compound against TrkB-dependent neuroblastomas, supporting the initiation of a phase 1 clinical trial for this compound in neuroblastoma patients with refractory disease [85]. In this case, the effectiveness of entrectinib in inhibiting neuroblastoma growth in vivo was determined after either single use of this compound or after its combination with the conventional chemotherapeutic drugs irinotecan and temozolomide. Given the frequency of *ALK* gene deregulation in neuroblastoma patients, it is reasonable to expect that many pharmaceutical companies will search for second-generation ALK inhibitors, possibly with more specificity for *ALK* mutations. Some of these inhibitors are in preclinical or clinical phases for neuroblastomas, such as LDK378 (ceritinib; Novartis Pharmaceuticals; NCT01742286 – Table 1) [69] and Alectinib (CH5424802; Alecensa; NCT01871805 – Table 1) [86].

A serious issue that remains is whether mentioned anti-ALK compounds would lead to the development of resistance, which was observed for crizotinib [87]. However, this seems not to be the case for PF-06463922, a potent and selective next-generation ROS1/ALK inhibitor tested by Infarinato et al. [88]. The authors described PF-06463922 as an extremely efficient drug when used for the treatment of neuroblastoma in crizotinib-resistant xenograft mice. The compound not only showed a potential to overcome crizotinib resistance but also a high capacity to induce complete tumour regression when administered alone in vivo. It should be emphasized that quicker detection of *ALK* mutations within tumour biopsies responsible for resistance to crizotinib would be necessary. Numerous ongoing investigations into the effectiveness of anti-ALK therapeutics provide confidence that we will soon be closer to a cure of HR neuroblastoma with deregulated ALK RTK.

### TrkA and TrkB: different roles in neuroblastoma

A line of evidence suggests that the TRK family of neurotrophin receptors plays a critical role in the diverse courses of neuroblastoma development. Human *TrkA* gene maps to 1q21, but no mutations or activating rearrangements have been identified in neuroblastoma [89]. Neuroblastomas are biologically favourable and susceptible to spontaneous regression or differentiation when TrkA is expressed. In this case, neuroblastoma fate depends greatly on the absence or presence of the TrkA ligand, nerve growth factor (NGF). In most tumours of patients in advanced stages, TrkA expression is low or absent, and such tumours do not undergo complete differentiation in response to NGF. This indicates that the NGF/TrkA pathway is responsible for differentiation and regression of favourable neuroblastomas. Another human Trk, *TrkB*, was cloned and mapped to 9q22 [90], and similarly, no mutations or activating rearrangements for this gene have been found in neuroblastomas to date. The TrkB receptor and its ligand are highly expressed in biologically unfavourable neuroblastomas. Full-length *TrkB* and *BDNF* are expressed in more aggressive neuroblastomas, and their expression is highly correlated with *MYCN* amplification [91]. In addition, it has been shown that TrkB expression in neuroblastomas is associated with drug resistance and expression of angiogenic factors [92]. Thus, the expression of both BDNF and full-length TrkB may represent an autocrine or paracrine survival pathway that is important for the aggressive behaviour of some neuroblastomas [93, 94]. Because TrkB has been correlated with poor outcome of neuroblastoma patients [95], its targeting in neuroblastoma is reasonable. GNF-4256, a selective and potent pan-Trk inhibitor (Novartis; Fig. 1b), is one of the compounds designed to target TrkB. This inhibitor demonstrated potent cytotoxic effects, both in vitro and in a mouse xenograft model [96], when used alone or in combination with irinotecan and temozolomide. These results suggest that GNF-4256 is an attractive compound for the therapy of relapsed neuroblastoma patients with dysregulated TrkB. Moreover, preclinical studies confirmed its low toxicity. Promising anti-tumour activity was also reported for AZD6918, a recently proposed novel pan-Trk inhibitor, that was tested in vivo [97]. Similarly to GNF-4256, AZD6918 showed strong inhibitory effects on tumour growth when used in combination with other conventional chemotherapeutics, such as etoposide. These results suggest that Trk (TrkB preferentially) inhibitors might be effective in personalised therapies for neuroblastoma patients with deregulated TrkB activity. A more detailed study in this field was performed by Nakamura et al. [98], who tested a series of synthetic candidate compounds predicted to have anti-TrkB activity *in silico*. These compounds were then analysed in vitro and in vivo to evaluate their efficiency against neuroblastoma tumour growth. The most efficient compounds identified in this study were suggested as drugs against TrkB-dependent neuroblastomas. Whether they might repeat their effectiveness in preclinical studies remains to be validated.

### Drugs against the PI3K/AKT/mTOR pathway

A recent study showed that the persistence of *ALK* mutations, and hence its constitutive activation, led to over-activation of several downstream signalling pathway, including PI3K/AKT/mTOR, in a subset of neuroblastoma [80]. Berry et al. showed that co-expression of one of the most common *ALK* mutations (*ALK* ^*F1174L*^) and MYCN amplification up-regulated several down-stream pathways, including the PI3K/AKT/mTOR pathway, in a neuroblastoma mouse model. In addition to ALK, several other RTKs and/or their ligands have been implicated in the increased activation of the PI3K/AKT/mTOR pathway in neuroblastoma [99]. However, although there is increasing evidence supporting a role of the PI3K/AKT/mTOR pathway in the development and progression of neuroblastoma, the molecular mechanisms that actually activate the PI3K/AKT/mTOR remain to be elucidated.

Certainly, it is to be expected that by blocking a part of this pathway, the proliferative capacities of neuroblastoma tumour cells should be inhibited. Still, the most relevant question that remains to be answered is where is the Achilles heel of this signalling cascade in tumour cells and where should we strike? Numerous inhibitors have already been developed, and some of them have been tested in neuroblastoma [100]. Because PI3K/AKT/mTOR pathway inhibitors have been discussed in many reviews already, e.g., Pal et al. [101], Mei et al. [102], we will focus only on the therapeutic aspects of the latest scientific reports.

A strategy involving the blockade of mTOR’s function to ameliorate ALK inhibition itself has been proposed by Moore and colleagues [87]. The authors observed that ALK inhibition by crizotinib did not affect all branches of the downstream pathways of ALK, leaving the mTOR-dependent signalling pathway active. The important relationship between ALK and the PI3K/AKT/mTOR pathway has also been illustrated by the finding that combined treatment with the ATP-competitive mTOR inhibitor Torin2 overcame the resistance of *ALK* ^*F1174L*^/*MYCN* tumours to crizotinib. In the same work [87], the authors combined crizotinib with mTOR inhibitors. This combination led to a strong cell cycle arrest and, importantly, prevented the growth of neuroblastoma tumours, suggesting that multiple attacks of ALK downstream pathways might be necessary for efficient defeat of tumour. Westhoff et al. [103] proposed similar experiments to improve effectiveness against neuroblastoma by using NVP-BEZ235, a PI3K/mTOR inhibitor (Fig. 1b), together with conventional chemotherapeutics. However, we must exercise caution in planning strategies against PI3K in the battle against neuroblastoma. As explained by Westhoff and colleagues [103], we must consider proposed drug use critically, keeping in mind that usually *“there is no linear link between degree of inhibition that we provoke chemically and inhibition of tumour growth”*. On the other hand, numerous studies have proposed the combined targeting of AKT with various biological agents as a more successful approach. There is a clinical trial (NCT01049841 - Table 1) ongoing for perifosine, which is one of the best-characterized AKT inhibitors, in combination with the mTOR inhibitor temsirolimus. It is expected that this combination would provide a better impact on tumour growth, ensuring a synergic effect between these drugs that has been observed in previous preclinical studies [104]. This therapeutic choice can be additionally justified by the results obtained from the clinical studies in which temsirolimus, used as mono-therapy, worked as cytostatic and guaranteed a stable disease after 12 weeks of treatment [105]. At the moment some clinical trials are recruiting patients to test temsirolimus in combination with standard chemotherapy and monoclonal antbodies, in order to seek for more promising cure of neuroblastoma patients with deregulated PI3K/AKT/mTOR signalling (NCT01767194 - Table 1). Whether neuroblastoma patients would benefit from these therapy remains to be seen.

### Drugs against the anti-apoptotic molecules - Survivin, BCL2 and HSP90

Survivin is another molecular biomarker whose enhanced expression was correlated with poor prognosis in neuroblastoma patients [106]. Encoded by the gene *BIRC5*, this protein has anti-apoptotic activity and represents an interesting druggable target whose blockage might provide significant benefits to HR neuroblastoma patients [107, 108]. Therefore, this candidate is an attractive target in neuroblastoma, even though its eventual integration in currently used therapy has not been considered profoundly. One of the compounds that regulates Survivin expression and hence its cell death-protective role is YM155 (Fig. 1c) [109]. The most important fact is that YM155 shows efficacy in eliminating tumour cells with acquired resistance to doxorubicin, vincristine and cisplatin. These findings imply that Survivin depletion could assure benefits to the patients in whom standard therapy has limited effects.

BCL2 is a protein with an important role in cell surviving [110, 111]. Although *BCL2* mutations are rare in neuroblastoma, this pro-survival protein plays an important function in neuroblastoma due to its deregulated expression [112, 113]. In fact, expression profiling studies have confirmed the increased levels of *BCL2* gene in many neuroblastomas. Therefore, BCL2 likely represents a good molecular target for neuroblastoma treatment. Several anti-BCL2 drugs have been designed to date (among which is a BH3 mimetic), such as ABT-263 and ABT-737, which appear to be particularly promising and efficient [114]. Nevertheless, the effect of the aforementioned inhibitors in neuroblastoma is still to be investigated sufficiently.

Recently, much attention has been paid to the inhibition of Heat shock protein 90 (Hsp90) as a strategy for neuroblastoma treatment. As a central molecule of complex folding machinery, HSP90 acts as a major regulator of protein integrity and function for the vast majority of proteins, including those with oncogenic potential [115]. High expression of HSP90 ensures protection from degradation for numerous proteins inside the cell, including ERBB2, AKT, MET and MYCN. Hence, over-expression of HSP90 protein in malignancies has been described as an anti-apoptotic feature, and its abrogation is seen as a therapeutic option even in neuroblastoma [116]. A role of HSP90 in protecting MYCN from degradation was observed when 17-DMAG (Alvespimycin), a small inhibitor against HSP90, was used in vitro. Interestingly, the same treatment also decreased the expression of AKT [117]. Another intriguing approach for targeting HSP90 in neuroblastoma has been proposed by Sidarovich et al. [118]. The authors discovered the potential to suppress the translational efficiency of heat shock proteins, including HSP90, by using compounds with iron-chelating characteristics. As a result, the authors observed a significantly reduced growth of neuroblastoma in a cell culture system. However, it is clear that additional work and clinical trials are necessary to evaluate whether the anti-apoptotic drugs can be a valuable clinical tool. In summary, although positive results from the preclinical testing of drugs against anti-apoptotic proteins have been obtained, it still remains to be seen if these drugs will be employed clinically as therapeutic strategies for the treatment of neuroblastoma.

## Current views and directions in neuroblastoma therapy: the Omics as the basis for personalised medicine

Among all Omics, the advent of massive parallel sequencing approach, so-called Next Generation Sequencing (NGS), has enabled a more detailed and deeper molecular characterisation of the neuroblastoma tumours. The analysis of the entire genome and exome showed genomic alterations associated with the molecular pathogenesis of neuroblastoma [119–124]. In particular, somatic point mutations and somatic structural variants in the *PTPRD*, *ODZ3*, *CSMD1* and *ARID1A* genes [120, 123], a few high-frequency recurrent somatic mutations in the *ALK*, *CHD9*, *PTK2*, *NAV3*, *NAV1*, *FZD1*, *ATRX*, *ARID1B*, *TIAM1*, *ALK*, *PTPN11*, *OR5T1*, *PDE6G*, *MYCN* and *NRAS* genes [119, 120, 122, 123] and rearrangements in *TERT* gene super enhancer region [121, 124] are discovered in neuroblastoma patients with worst survival.

Considering all currently available genomic data, several national and international groups operating in neuroblastoma field discussed in March 2015 during the SIOPEN Genomics Meeting in London, a NGS neuroblastoma signature for tumours of HR patients. At this meeting the collaborators proposed a panel of mutations, determined by whole exome sequencing (WES), to be screened in neuroblastoma patients, defining in that way a NGS signature specific for neuroblastoma [70]. The use of NGS profile is the first step towards personalised medicine in this paediatric malignancy. Subsequently, genomic data assisted in the development of pharmacogenomic technologies that allow the determination of specific therapeutic approaches for genetically homogenous cohorts of patients. It is expected that the current therapeutic protocol adopted for patients of one risk group will be replaced by a specific drug combination designed to treat patients based on their specific genetic profiles. A pioneer result that compare mutational spectrum in mitochondria (mt) versus nuclear (n) DNA in neuroblastoma patients at diagnosis and at relapse has been published by Reihl et al. [125]. To address the question if and in which extent DNA appertaining to these two cell compartments varies at spatiotemporal scale they applied WES. They found that both mtDNA and nDNA showed similar variations in relapsed samples with respect to samples obtained at diagnosis. Hence, the authors suggest that observed genetic variances could be useful biomarkers for monitoring of neuroblastoma progression. In support to this concept, recent studies on matched primary tumours and biopsies at relapse clarified that genetic alteration in *CHD5*, *DOCK8*, *PTPN14*, *HRAS* and *KRAS* genes and losses on chromosome 9p acquired during tumour progression suggesting a likely tailored therapy against these genetic alterations in patients at the disease recurrence [126]. Furthermore, the authors showed that the overall count of mutations in biopsies at relapse is higher than in primary tumours. In another independent, non-overlapping study, 78% of recurrent tumours harboured a higher overall mutations count compared to primary tumours showing an hyperactivated RAS-MAPK signalling pathway [127]. Both reports introduced the concept of temporal and dynamic cancer model in which neuroblastoma primary tumours were composed of a minor population of multiple clones that persisted throughout the therapy, expanding then at the recurrence [128]. Together, these studies suggest that the analysis of recurrent tumour biopsies is mandatory for any clinical trial [128].

### Metabolomics and proteomics – is it time to move therapy towards precision medicine?

Additional Omics that will certainly contribute to more effective personalised medicine are metabolomics and proteomics. The analysis of small-molecule metabolites is an advantageous means to differentiate normal from malignant tissue and to predict tumour treatment response [129–131]. Indeed, Imperiale and colleagues [132] defined a metabolite profile using tumour of neuroblastoma patients, establishing differences in their profiles with respect to healthy tissues. More precisely, they defined the so-called metabolic fingerprint of neuroblastoma as a metabolic marker to control the disease course. Another valuable approach includes metabolome analysis of patients’ sera to improve the reliability of diagnosis or risk-stratification of neuroblastoma patients, as reported by Beaudry et al. [133]. The authors performed a retrospective metabolome study, examining whether the patient’s sera discriminate low from HR neuroblastoma patients. They observed equally distributed metabolite profile between low and HR patients using nuclear magnetic resonance (NMR). In addition, they analysed metabolites profile in sera of mice after neuroblastoma xenografts by NMR and gas chromatography–mass spectrometry. Importantly, they distinguished the metabolites differentially present at early phase versus late stage of disease proposing them as possible biomarkers to determine a presence of early stage tumours. Moreover, the results of these analyses done in humans and repeated using sera of xenografted-mice gave comparable profiles confirming that the xenografts recapitulate the behaviour of human tumours. These observations imply that the analysis of metabolome profile from neuroblastoma patients’ sera, together with other diagnostic tools already used in clinic, could enable more accurate prediction of tumour behaviour. In any case, at this moment larger studies are needed to determine whether identification of key metabolites in patients’ sera can be used as diagnostic tools in neuroblastoma. As far as proteomics is concerned, the level of specific protein biomarkers in the plasma of neuroblastoma patients can determine HR neuroblastoma [134]. These results support the integration of proteomic approaches as fast and non-invasive techniques in the monitoring of neuroblastoma behaviour in HR patients. Additional findings that provide evidence in favour of metabolic markers have been provided by Otake et al. [135], who defined new biomarkers of an unfavourable neuroblastoma phenotype, applying shotgun proteomic analysis. The authors focused particular attention to the protein DDX39A, which might be considered a novel marker for proteomics approaches to HR neuroblastoma diagnosis. Several in vitro validation studies also gave encouraging data that a proteomic approach can be applied to define the diverse intracellular pathways and molecules that are responsible for: i) an aggressive neuroblastoma phenotype or ii) resistance to therapy [136, 137].

### High-throughput drug screening

The National Cancer Institute has launched a program to assess new drugs for paediatric use, called the Paediatric Preclinical Testing Program (PPTP) [138]. The PPTP is an initiative to identify therapeutic drugs that have significant activity against childhood cancers, including neuroblastoma. The PPTP has established panels of childhood cancer cell lines and xenografts to be used for in vitro and in vivo testing. The PPTP has the capacity to test approximately 12 compounds or combinations of compounds in preclinical models of childhood cancers. The cancers include Wilms tumour, sarcomas (rhabdomyosarcoma, Ewing sarcoma and osteosarcoma), neuroblastoma, brain tumours (glioblastoma, ependymoma and medulloblastoma), rhabdoid tumours (CNS and renal) and acute lymphoblastic leukaemia (ALL). The selection of drugs for PPTP testing is based on their potential relevance in the childhood cancer setting and their stage of clinical development. In parallel, standard drugs are also being tested, both to calibrate the PPTP tumour panels and to serve as a basis for future combination studies [107]. Between 2008 and 2015, more than 60 reports of initial testing (Stage 1) were published by the PTPP. From the point of the in vitro studies, another interesting approach arrives and proposes high-throughput screening for the best single or combined drug selection. In fact, an increasing number of reports identified high-throughput screening as useful methodology to select additional functional anti-tumour drugs. Indeed, an example is the screening of compounds against the neuroblastoma cell line IMR32, from which it was discovered that the *PHOX2B* gene might be targetable by influencing its direct transcriptional regulators, such as Meis-1, NF-κB and AP-1 [139]. Accurate evaluations of high-throughput screening in neuroblastoma have been described by Harder et al. [140]. Therefore, we propose that introducing this technique could lead to increased identification of promising compounds for neuroblastoma treatment. The identification of new compounds could allow us to increase the number of clinical trials for personalised medicine.

### Epigenetic biomarkers and regulatory RNAs

Recently, analysis of epigenome profiling and microRNA (miRNA) expression patterns performed in neuroblastoma samples has provided a significant amount of data, identifying the targeting of epigenetic regulators as a possible treatment strategy. It is also expected that epigenomic studies will identify new biomolecular markers that may lead to a better stratification of neuroblastoma patients.

#### Epigenetic background of neuroblastoma

Aberrant DNA methylation, either hyper- or hypo-methylation, has emerged as a new hallmark of tumourigenic processes [141]. In particular, changes of the “physiological” methylation patterns have been correlated with neuroblastoma patients’ prognosis [142]. Additional studies of DNA methylation profiles in neuroblastoma tumours have identified the pro-apoptotic gene *CASPASE 8* and the tumour suppressor gene *RASSF1A* as novel target molecules. The hyper-methylation of their promoter regions, and hence reduced or absent gene expression, has been confirmed in the majority of examined neuroblastoma [143]. Soledad Gómez and colleagues revealed that major DNA methylation changes took place outside promoter regions. More importantly, they observed that the changes in the methylation pattern are associated with clinico-pathological characteristics of neuroblastoma [144]. A similar conclusion was drawn by Buckley et al. [145], who associated a hyper-methylation pattern with diverse neuroblastoma phenotypes.

#### Non coding RNAs

Another class of biological molecules whose expression depends on epigenetic regulators are microRNAs (miRNAs). As non-coding RNA molecules, miRNAs are able to control the expression of genes at the post-transcriptional level. miRNAs have emerged as very important biomarkers of many cancers including neuroblastoma. In fact, an increasing number of studies indicate that imbalanced expression of miRNAs could offer an alternative explanation for neuroblastoma aggressiveness and serve as a basis for selection of more efficient drug combination [146]. Even at this level MYCN is an important player, since some miRNAs are described as direct transcription targets of MYCN. Among them, several miRNAs with tumour-suppressor features (e.g. miR-184, miR-181a-5p, miR-181b-5p, miR-320a) [147] are evidenced. These findings suggest that MYCN, beside direct impact on its target genes, can indirectly regulate a subset of other genes at post-transcriptional level. There are several data that indicate that miRNAs profiles are predictive for the outcome of neuroblastoma patients [148–150]. Some of the suggested miRNAs might be interesting targets to be combined with standard therapeutic protocols for neuroblastoma cure in future. High throughput studies of long non-coding RNAs (lncRNAs) also highlighted the role of these regulatory RNAs as promising drug targets for therapeutic interventions. Indeed, a recent sequencing transcriptomes analysis of low- and HR neuroblastomas pinpointed a lncRNA neuroblastoma associated transcript-1 (*NBAT-1*) as a biomarker that predicted neuroblastoma patients outcome [151]. The authors showed that *NBAT-1* was necessary for differentiation of neuronal precursors and that hypermethylation of its promoter region and following gene down-regulation increases neuroblastoma cells proliferation. Being described as tumour suppressor, *NBAT-1* might be among crucial regulatory RNAs and so, the therapy against *NBAT-1* and its downstream effectors could be a potential novel therapeutic option for the treatment of HR neuroblastoma [151]. Moreover, another recent study evaluated differential expression profiles of lncRNAs and protein-coding genes between *MYCN* amplified and non-amplified neuroblastomas by examining microarray and RNA-seq datasets [152]. The authors revealed correlation between *SNHG1* regulation and *MYCN* amplification and suggested *SNHG1* as another indicator of neuroblastoma patient’s outcome and/or as an option for therapeutic targeting.

## Liquid biopsy as a useful technique for Omics studies

**Fig. 2:**
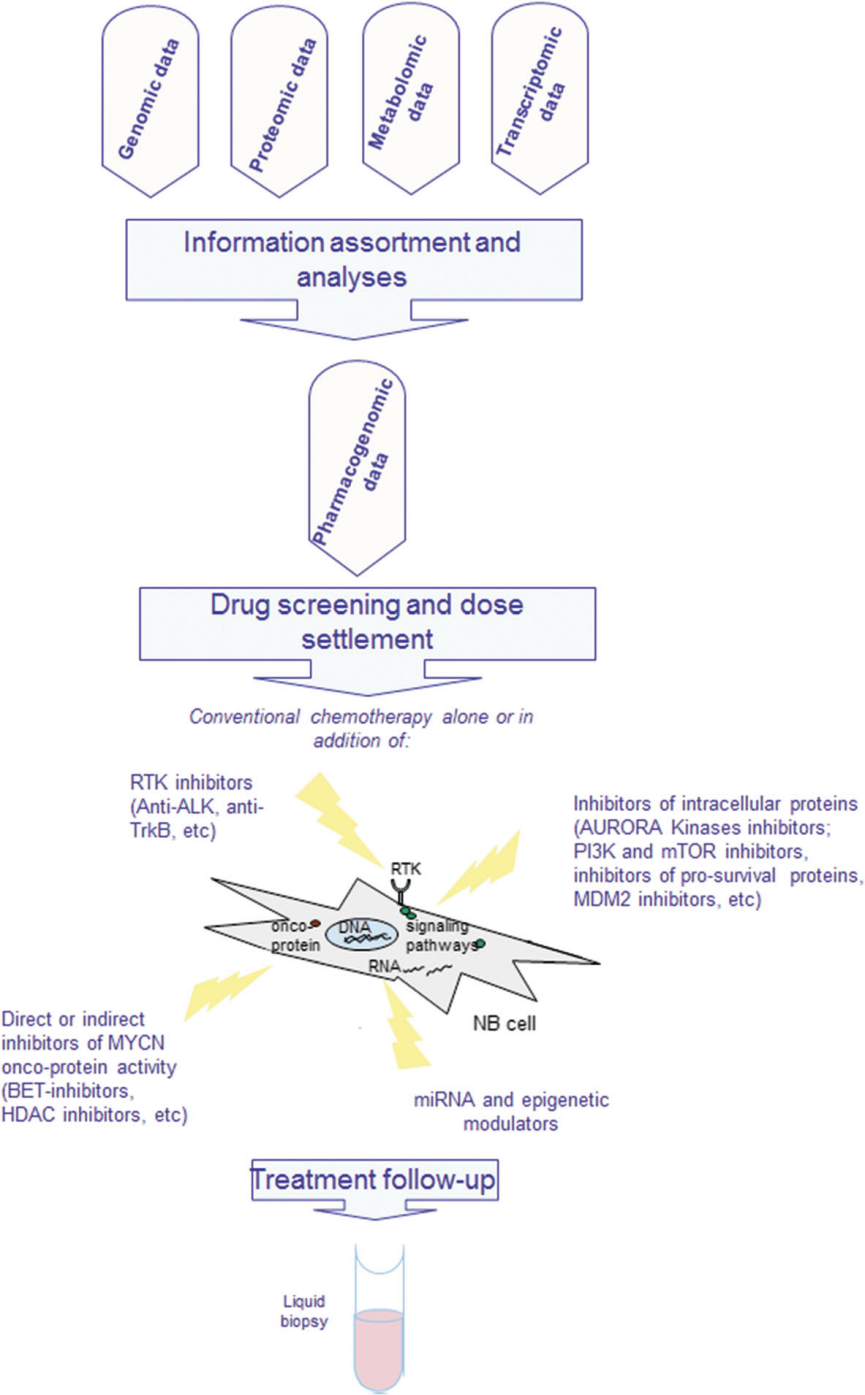
Flow chart of Omics data integration for personalised treatment. At the top of the figure, schematic presentation of Omics data processing, the assortment and analyses is shown. Once collected, omic technologies data obtained by studying tumor material or CTC from blood, have to be integrated to allow the extraction and selection of the druggable single target or molecular pathways. This step proceeds by the screening of the most reliable drug or drug combination that would assure optimal chances for tumour defeating. Some of the compounds are listed in the figure: MYCN, ALK, AURORA A, TrkB. In the lower part of the figure is shown the liquid biopsy as a procedure to use at the follow-up in order to understand how disease behaves due to treatment. In the case of neuroblastoma, integration of liquid biopsy for the follow-up of molecular biomarkers during therapy might be a winning strategy for early detection of possible drug resistance that could allow clinicians to change current therapeutic strategy

In recent years, liquid biopsy has been used as a biological sample that enables diagnosis and monitoring of disease status. Blood liquid biopsy contains circulating tumour cells, which can be studied by Omics technologies. In neuroblastoma, liquid biopsy is very useful, particularly if tumour cells are not available from the primary tumour or are not sufficient for genomic studies. The importance of liquid biopsy for tumour studies was realised when it is discovered that the levels of cell-free circulating DNA (ctDNA; small, double-stranded fragments of DNA) in the blood were much higher in patients with cancer than in those of healthy individuals. These findings provide a basis for the possibility of using liquid biopsy to monitor the disease in a simpler and faster way [153]. In neuroblastoma, liquid biopsy is useful in evaluating *ALK* and *PHOX2B* mutations, *MYCN* expression etc. As far as neuroblastoma is concerned, some research groups have already begun testing whether liquid biopsy could mirror the genetic profile obtained directly from tumours [154, 155]. The first data obtained by the use of liquid biopsy at neuroblastoma diagnosis are promising. They suggest that this non-invasive approach could serve as a detection method of neuroblastoma at early time points of disease, allowing an immediate and suitable treatment. Liquid biopsy is also useful for the evaluation of the response to therapy by allowing for more frequent follow-up measurements of specific molecular markers persisting in liquid biopsies. A diagram of the workflow of liquid biopsy use in neuroblastoma and its integration with Omics technologies is shown in Fig. 2. Because the clinical utility of this approach has been already demonstrated for other types of cancer [156], its use in neuroblastoma provides new prospects for more accurate and faster diagnosis of the disease and eventual determination of the correct approach in personalised therapy.

## Conclusions

In this review we provided an update of the pharmacological achievements proposed for neuroblastoma treatment during the last years. Moreover, we discussed the way that neuroblastoma management could be influenced by the findings obtained from Omics technologies. We also reported the importance of high-throughput Omics data for better risk’s stratification of the neuroblastoma patients in order to provide a more efficient targeted therapy. From these techniques, it is expected to allow, in a rapid and strict way, a generation of the list of possible druggable targets that clinicians should consider to improve patients health. Indeed, these techniques will lead to the increased number of successful clinical trials which will contribute to increasing cure rates of neuroblastoma patients.

In conclusion, we believe that the use and integration of Omics data within other clinical and biological information already defined for neuroblastoma is the right direction toward precision medicine. Under this scenario, the Omics profiles should allow a more accurate characterisation of neuroblastoma phenotype providing a support for an efficient therapy against this pediatric tumor.
